# Improving the data access control using blockchain for healthcare domain

**DOI:** 10.12688/f1000research.72890.2

**Published:** 2021-10-27

**Authors:** Olaosebikan Tahir Yinka, Su-Cheng Haw, Timothy Tzen Vun Yap, Samini Subramaniam

**Affiliations:** 1 Faculty of Computing & Informatics, Multimedia University, Cyberjaya, Selangor, 63100, Malaysia; 2 AirAsia Berhad, KLIA2, Selangor, 64000, Malaysia

**Keywords:** Blockchain, Healthcare datasets, Chaincode, Directed Acyclic Graph, Access Control.

## Abstract

**Introduction: **Unauthorized access to data is one of the most significant privacy issues that hinder most industries from adopting big data technologies. Even though specific processes and structures have been put in place to deal with access authorization and identity management for large databases nonetheless, the scalability criteria are far beyond the capabilities of traditional databases. Hence, most researchers are looking into other solutions, such as big data management.

**Methods: **In this paper, we firstly study the strengths and weaknesses of implementing cryptography and blockchain for identity management and authorization control in big data, focusing on the healthcare domain. Subsequently, we propose a decentralized data access and sharing system that preserves privacy to ensure adequate data access management under the blockchain. In addition, we designed a blockchain framework to resolve the decentralized data access and sharing system privacy issues, by implementing a public key infrastructure model, which utilizes a signature cryptography algorithm (elliptic curve and signcryption). Lastly, we compared the proposed blockchain model to previous techniques to see how well it performed.

**Results: **We evaluated the blockchain on four performance metrics which include throughput, latency, scalability, and security. The proposed blockchain model was tested using a sample of 5000 patients and 500,000 observations. The performance evaluation results further showed that the proposed model achieves higher throughput and lower latency compared to existing approaches when the workload varies up to 10,000 transactions.

**Discussion: **This research reviews the importance of blockchains as they provide infinite possibilities to individuals, companies, and governments.

## Introduction

The US social insurance system, such as healthcare, is gradually obtaining digital medical information, thanks to the fast-expanding availability of data which will considerably build up the number of medical information collected securely. ^1^ Furthermore, a change in perspective in the medical services sector has seen the new trend of computerizing medical records. The medical services sector is therefore rapidly rising in data volume as far as randomness and simplicity are concerned. Although the public healthcare sector is struggling with the magnitude of big data, security and privacy concerns are converging as threats and vulnerabilities grow. ^2, 3^

The modern and revolutionary technology to build safer, more private, and interoperable health systems, blockchain technology has proven to be efficient. The aim of this paper is to propose a decentralized model for sharing and storing medical information utilizing blockchain technology to ensure security, preserving complete privacy, and allowing the owner to be completely in control of their data. In this research, additional efforts were made to harness and itemize all blockchain technology technical tools and the current influence of this developing technology on healthcare and industry.

Recent studies in the healthcare sector dealing with is-sues of scalability have used blockchain. Nonetheless, the application of blockchain in these studies was limited to the healthcare dataset index and the storage of verified transactions by endorsing peers. In contrast to these approaches, the current blockchain model pro-poses the storage of all medical datasets in blockchain and also improving data throughput limitations. In light of these proposed improvements, Yang *et al.* ^4^ proposed the use of blockchain as an index of a medical dataset and a list of user’s information. Similarly, Daraghmi *et al.* ^5^ introduced a data-sharing and access authentication scheme based on a private blockchain. Likewise, Genestier *et al.* ^6^ offered a novel proposal for reshaping healthcare permission monitoring using blockchain technology and allow users to access their entire health record data. In their implementation, there is no permission settings and no access restriction. MedRec ^7^ is a software system developed using blockchain technology by the MIT media lab to handle electronic health records. They created a hospital management system that responds to four major issues: disjointed, delayed access to health care information; process compatibility; respect for autonomy; and enhanced data quantity and quality for clinical research.

Medical Chain ^8^ project began in 2016, with the launch of the first model in mid-2017. Later in 2017, a partnership with the Linux Foundation was created. In the early months of 2018, a beta version of the program was published. The primary objective is to develop a single, accurate version of the user's health data.

MedBlock, ^9^ which is architecture based on a hybrid blockchain to protect Electronic Medical Records (EMRs). Their design nodes identify them as endorsers, orderers, and committers. Yet, the access control strategy to allow third-party researchers access to medical data is not specified precisely.

Xia *et al.* ^10^ introduced the Blockchain-Based Data Sharing (BBDS) system allowing owners and participants to access EMR from a shared repository theory based on positive identity and key verification.

Diversely, other research projects related to adopting blockchain in the healthcare domain are summarized as follows.

Blockchain Health, ^11^ ASTRI ^12^ and Bloq ^13^ allow users to use the integrated platform to share health data with researchers, which creates a tamper-proof knowledge custody chain. Similarly, HIE of One ^14- 16^ is a blockchain software initiative that provides resources for patients to access their own records. Hyperledger ^17^ and IBM Blockchain ^18^ is hyperledger fabric's first regulated platform, allowing the development of company blockchain platforms that users can monitor and spread through various organizations.

All the principal functions of blockchain technology and some true blockchain applications in healthcare were discussed at this point. More concretely, issues addressed in this chapter include authentication management, identity management, and cryptography. In the next section we will clarify the model that helps to il-lustrate how the blockchain network communicates with the developed model.

The paper contributions are summarized as follows:

1. A file storage and sharing system for a pure decentralized network utilizing blockchain technology is proposed.
2. An extensive evaluation of the proposed system, which demonstrated that our proposed system achieves higher throughput and lower latency compared to some existing approaches at higher workload varied up to 10,000 transactions.

## Methods

**Figure 1.**
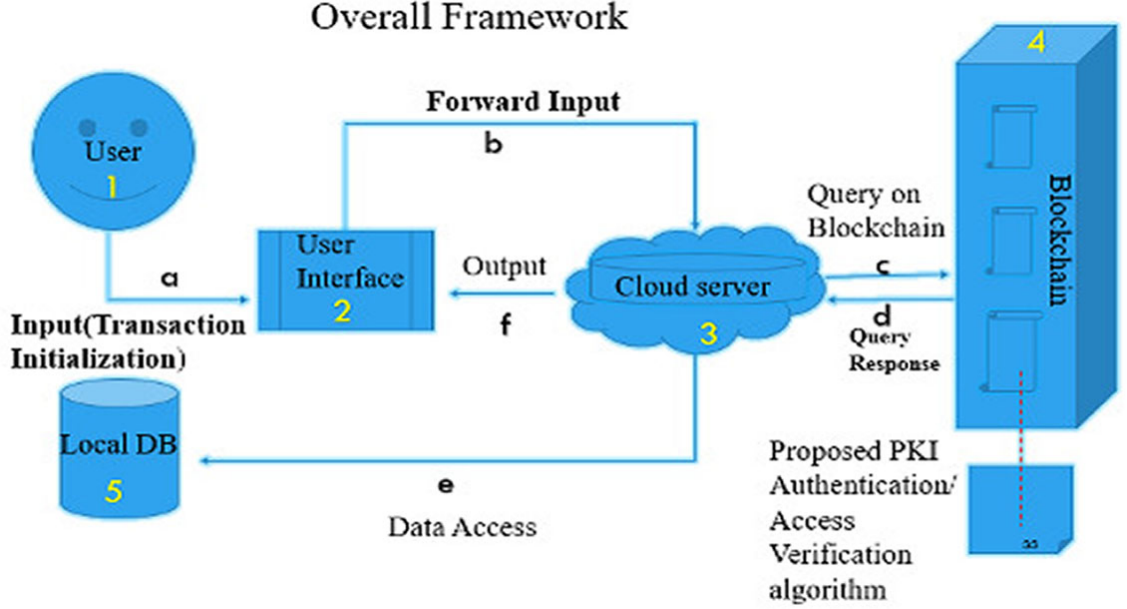
Overall framework of proposed system.

The overall data storage workflow is displayed in Figure 1 based on the following steps:

(1) A user joins through the graphical user inter-face (GUI) and the request for permission to access information can be inserted into the network when successfully authenticated.
(2) The user's request is sent to the cloud server by the GUI.
(3) The blockchain component, the server query enabled, or revoked access.
(4) The blockchain component handles content verification, signing, and encryption via Public Key Infrastructure (PKI).
(5) Finally, data updated/stored with the authenticated token takes place.

From Figure 1, steps (a) to (f) depict the procedures for accessing a data source:

(a) A user authenticates using the GUI. Upon successful authentication, user input will be taken into the system.
(b) Through the GUI, user input is forwarded to the cloud server.
(c) The cloud server then run queries against the blockchain for the user's access token by running the proposed PKI algorithm.
(d) The access token retrieved is been decrypted with the appropriate user keys.
(e) Data access is granted and retrieved with the help of a database connector.
(f) The server returns updates to display data in a format that is readable to the user.

### Case study: implementing the proposed model prototype to healthcare

We first analyzed a case study concerning the healthcare domain. We will then describe each of the key components in the case studies: (i) join function, (ii) content authentication function, and (iii) secure content access function.

The whole scheme works as follows. Consider a healthcare scenario of file storing and sharing. For each transaction, the ‘patient’ provides the contents to be stored and accessed. The ‘hospital administrator’ and ‘doctor’ request access to these contents. We assume that ‘hospital administrator’ and ‘doctor’ use our proposed model (see Figure 1), acting as the search mechanism to engage in the process of searching for content.

**The join function:** Let us consider a contrary of the scenario described above, if the doctor has no clearance of access, the doctor is, therefore, unable to access the desired content. In this case, by initiating the join function ( Algorithm 1), the content provider can update the doctor's clearance or refuse the proposal.

As shown in Algorithm 1 below, the identity is made up of signature key-pairs marked as SK for Bob and Alice, with reference pointers to the keys represented by RPSK. A public key denoted as PK is used to encrypt (and decode) the data, ensuring that the data is secured from all other players in the system.

**Algorithm 1. Join function (creating identity).**

1: **procedure** JOINFUNCTION (Alice, Bob)
2:  Alice executes:
3:     SK^Alice^ ← RP^Alice^
4:     EncRPSK^Alice^ = enc (PK^Bob^, RPSK^Alice^)
5:     Alice shares EncRPSK^Alice^ with Bob
6:  Bob executes:
7:     PK^Bob^ ← RP^Bob^
8:     Bob shares PK^Bob^ with Alice
9:  **return** EncRPSK^Alice^
10: **end procedure**

**The content authentication function:** In the same scenario described earlier in the join function, once the ‘user’ has access to the desired content, the ‘user’ must verify their authenticity with the content authentication function, this is done on the blockchain ( Algorithm 2).

**Algorithm 2. Content authentication against blockchain.**

1: **procedur**e POLICYCHECK (PK^K^, x_p_)
2:  Bob ← False
3:  a_policy_ = H (PK^K^)
4:  **if** M [a_policy_] ≠ **null then**
5:  PK^Alice^,0 PK^Bob^, POLICY_Alice,_Parse(M [a_policy_])
6:      **if** PK^K^ = PK^Alice^ **or**
7:  (PK^K^ = PK^Bob^ and x_p_ ∈ POLICY^Alice, Bob^) then
8:  Bob ← True
9:      **end if
**10:  **end if**
11: **end procedure**

Parse(x), also known as xp, is an auxiliary function that de-serializes a transaction's message, which includes the parameters. As seen in Algorithm 2, POLICYCHECK (PKK, xp) checks that the originator has the proper permissions. Memory on the blockchain, indicated by the letter “M”: We'll call M the blockchain memory space, which can hold suitably big documents and is represented by the hashtable L: {0, 1}256 → {0, 1} N, where N >> 256.

**The secure content access function:** Consider a situation where a ‘user’ wishes to use our model to access the desired content. The built model includes access chaincodes that record all ‘user’ activities on the blockchain server, such as record of shared data or records of information access history. Similarly as proposed by, ^19^ Algorithm 3 is executed when read/write transaction access is requested. Algorithms 3 and 4 runs on the same evaluation PC to ensure fair performance evaluation.

**Algorithm 3. Content access function (read/write function).**

1: **procedure** RWTX (PK^K^, m)
2:  c, x_p_, rw = Parse(m)
3:  **if** PolicyCheck (PK^K^, x_p_) = 1 **then
**4:     PK^Alice^, PK^Bob^. POLICY^Alice, Bob^ ← Parse (M [H () PK^Alice^])
5:     a_xp_ = H (PK^Alice^||x_p_)
6:  **if** rw = 0 **then**
7:        hc = H(c)
8:        M [a_xp_] ← M [a_xp_] ∪ hc
9:        (DHT) ds [h_c_] ← c
10:        **return** h_c_
11:     **else if** c ∈ M [a_xp_] **then**
12:        (DHT) **return** ds [h_c_]
13:     **end if**
14:  **end if**
15:  **return** 0
16: **end procedure**

As described earlier in this section, this architecture was designed for secure, scalable storage and data sharing in healthcare. The hyperledger network system is a permissioned blockchain implementation, providing unique properties suitable for implementing our proposed model. It runs chaincodes written in Java and Go programming language. Similar to Shadab et al., 2021 ^20^ the proposed model also supports data transaction validation applications and a plug-in consensus validation protocol. It is also important to note that hyperledger fabric collaboration network utilized consensus validation entities of a different kind, such as clients, ordering service nodes, and peer nodes, which belong to various participating entities or organizations.

## Experimental evaluations and results

In the evaluations, all algorithms run on the same PC, we observed the various experimental evaluations conducted for Hyperledger fabric. In the experiment, we considered scenarios where a client or user sends ‘N’ transactions of a certain type ‘f’ to the blockchain in an asynchronous manner. Between 1000 to 10,000 transactions were asynchronously sent to the blockchain server. The number of run transaction requests is set from 1000 to 10,000 transactions, with increments of 1000 transactions for every run a sample digitized data collection, namely OpenMRS that consists of 5000 patients and 500,000 observations is used for our evaluation. We have study latency and throughput up to 600blocks in the thesis presented for the work (see Table 1). We benchmarked the proposed model against approaches proposed by ^4, 5^ for a fair comparison.

**Table 1.** Experiment parameters.

Evaluation parameters	Evaluation values
Transactions/Queries submitted	1000, 2000, 300, 4000, 5000, 6000, 7000, 8000, 9000 and 10,000
Block sizes	200, 400, 600
Transaction in byte	1,000–10,000
The stored health records	10,000–100,000

### Case study: implementing the proposed model prototype to healthcare

**Figure 2.**
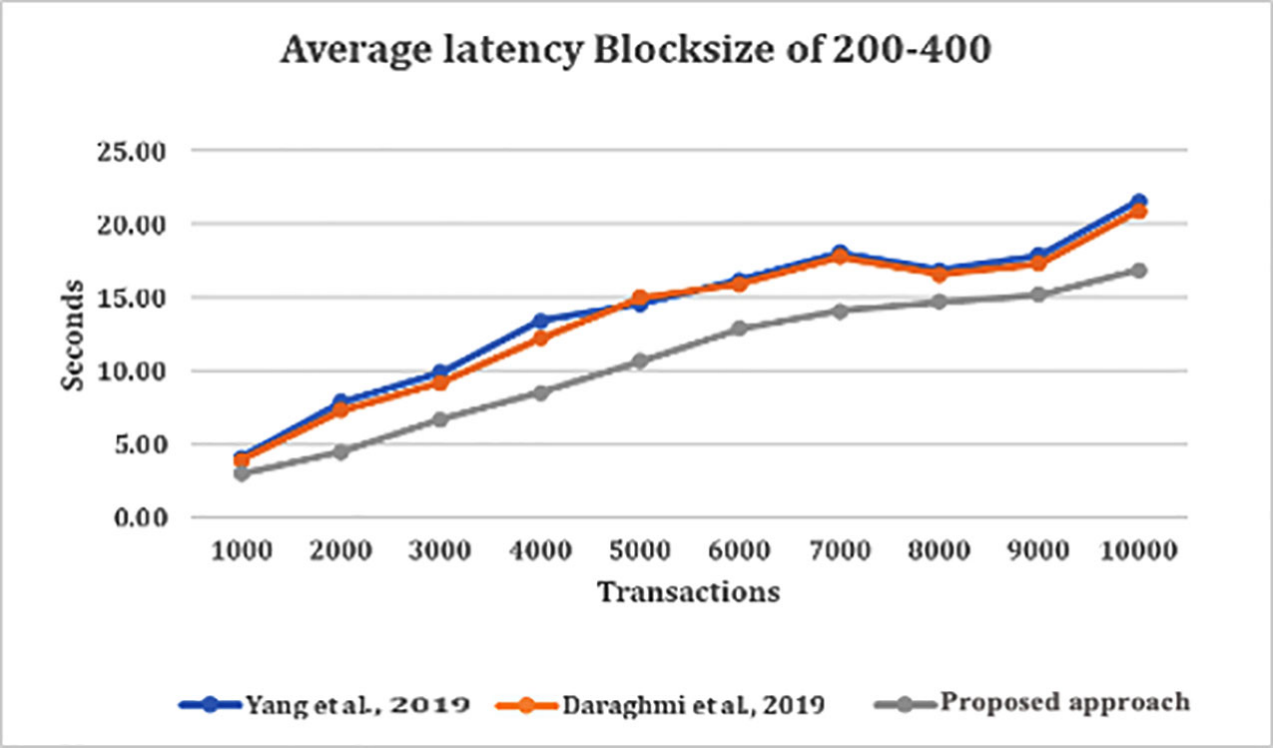
Average latency with block size of 200–400 on various transaction arrival rates.

Figure 2 depict the average latency across specific block sizes, analyzing various datasets or arrival rates of transactions. From Figure 2, we made several observations. This include the average latency in our approach is almost two-times less than that of average latency in both approaches by ^4, 5^ for all block sizes, except for when the transaction is at 1000 TX, we saw that latency of all approaches are lower. This is because the volume of pending transactions at validation stage grew on high count which affected commit latency.

### Evaluating the impact of endorsement policy

**Figure 3.**
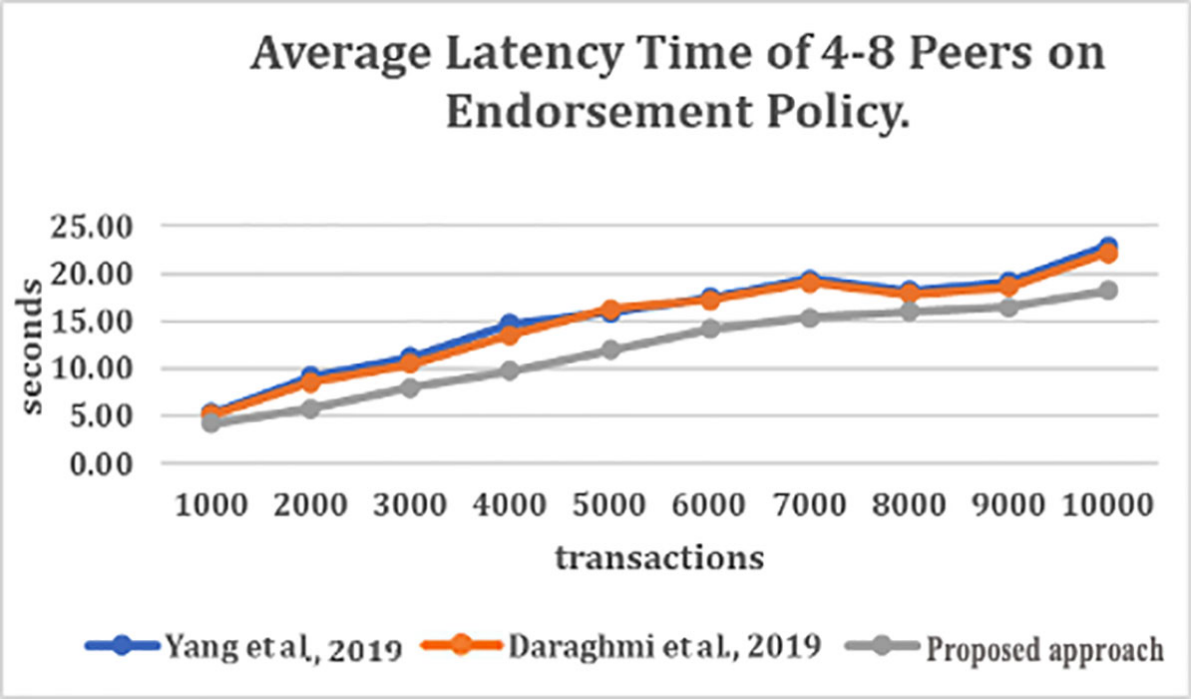
Average latency time of 4–8 peers on endorsement policy.

Figure 3 depict the average latency time of endorsement policy with the various number of transactions per second (TPS) (for the number of transactions ranging from 1000 to 10000). From Figure 3, it can be observed that the number of sub-policies and the number of signature validation has caused high latency for the join, content authentication and secure content access functions in ^4, 5^ approaches.

### Impact of state database

We study the throughput and latency over varying transaction arrival rates for database utilized in the three approaches with different transaction complexity.

**Figure 4.**
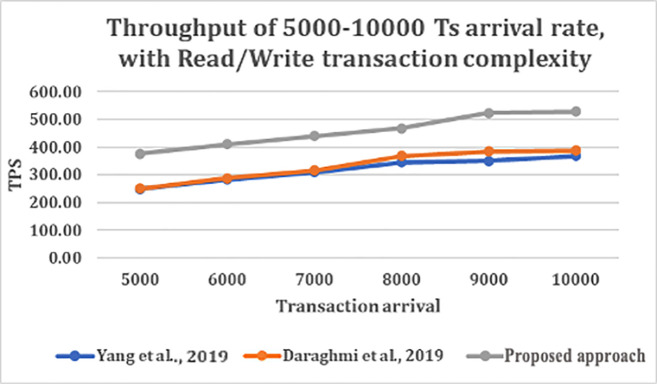
Throughput of 5000–10000 transaction arrival rate with read/write transaction.

**Figure 5.**
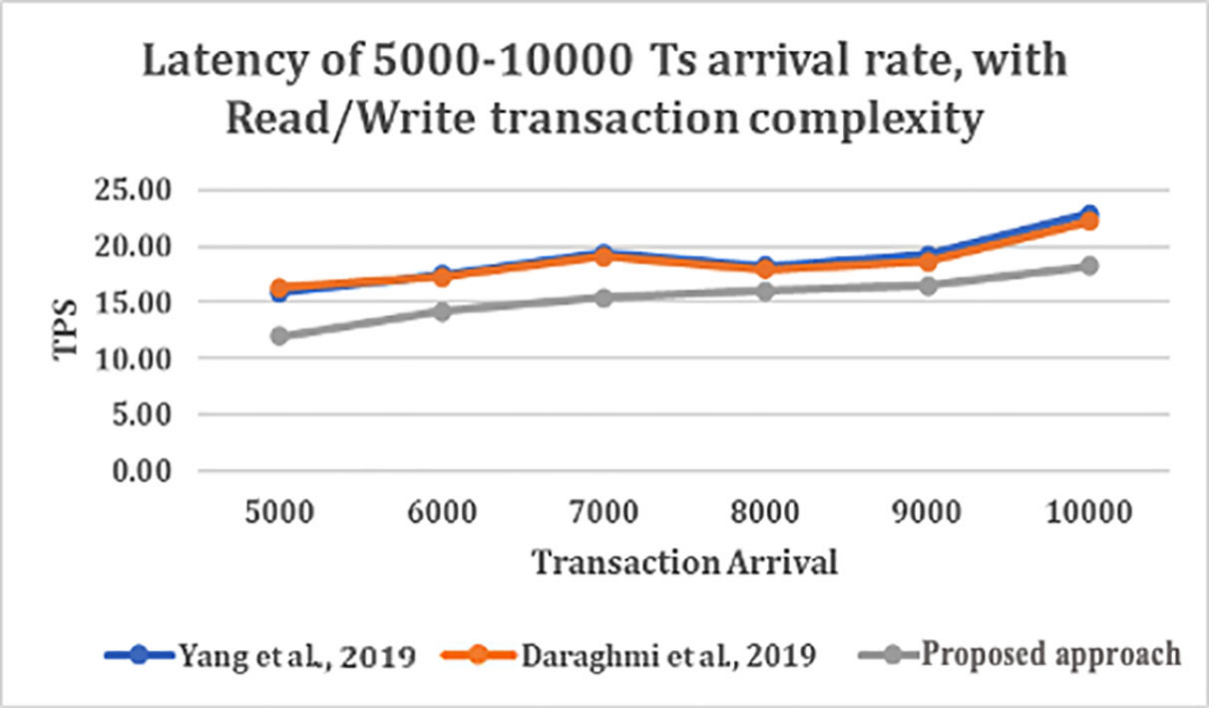
Latency of 5000–10000 transaction arrival rate with read/write transaction.

From Figures 4 and 5, shows that as transaction arrival increased for read/write only complexity, the throughput increased and latency decreased. This was observed while utilizing CouchDB as the state database.

The result shows that GoLevelDB performance was better than CouchDB with respect to ledger database. The reason behind the relatively good performance is the existence of key-value state database embedded in the peer mechanism.

## Discussion

The performance evaluation results further showed that the proposed model achieves higher throughput and lower latency compared to existing approaches when the workload varies up to 10,000 transactions.

It's also worth noting that the proposed model preserves confidentiality and security by controlling transactions with timed-based chaincodes. The use of sophisticated encryption maintains security and access control.

Security and access control are maintained by the adoption of advanced encryption (such as the proposed hybrid PKI) and authentication methods that defend against adversarial efforts to change unwanted entry when issuing encryption information to verify and validate transactions to network users.

## Conclusions

We had presented a model, which describes the interaction between a blockchain network and the designed model. To ensure a safe and private environment to communicate and store health data, the designed model exploits the most significant elements of blockchain technology while suppressing features that are not significant. In future work, we expect to perform refined simulations so that our model is tested more rigorously. We could do this by downloading and testing a wider range of various blockchain frameworks in a verified setting, for instance, using the blockchain instance of Amazon Web Services' blockchain.

## Author contributions

Olaosebikan Tahir Yinka constructed the protytpe and conducted the experimental evaluations under the guidance of his supervisors, Su-Cheng Haw and Timothy Tzen Vun Yap. Samini Subramaniam is the external collaborator while Su-Cheng Haw is the corresponding author for this paper.

## Data availability

No data are associated with this article.

## Software availability

• **Source code available from:** https://github.com/captaintaheer/Solution_Code
• **Archived source code at time of publication:** https://doi.org/10.5281/zenodo.5226652 ^21^
• **License:** Data are available under the terms of the Creative Commons Attribution 4.0 International license (CC-BY 4.0).

## Ethics approval

Ethical approval was given by the ethical approval board of Multimedia University, with number: EA1762021.
