# Supplementary material for: Improving the data access control using blockchain for healthcare domain
Source: F1000Res. 2021 Nov 19;10:901. Originally published 2021 Sep 8. [Version 3] doi: 10.12688/f1000research.72890.3 (PMC8579480; doi:10.12688/f1000research.72890.3)
Supplement: Peer review history

# Peer review: Reviewer response for version 2

**DOI:** 10.5256/f1000research.78587.r97373

**Published:** 2021-11-09

**Authors:** Heru Agus Santoso

**Affiliations:** 1 Faculty of Computer Science, Dian Nuswantoro University, Semarang, Indonesia

The paper intended to show the advantage of blockchain to improve data access control for the healthcare domain. The goal is to provide a blockchain framework to resolve decentralised access and authentication control. However, in my opinion, the paper needs some clarifications in regards to data analysis and text:

1. You said Algorithm 3 and 4 run on the same evaluation PC, but I didn't find Algorithm 4 in the text.
2. Algorithm 3 hasn't been explained adequately, especially in lines 4-5; and returned values in lines 10 and 12, what is the difference between the two?
3. In the sense of statistical analysis and interpretation, you said the latency was getting lower because the volume of pending transactions grew high? This needs a clear explanation.
4. Add the point of blockchain framework to resolve decentralised access and authentication control in the discussions.

Is the work clearly and accurately presented and does it cite the current literature?

Yes

If applicable, is the statistical analysis and its interpretation appropriate?

Partly

Are all the source data underlying the results available to ensure full reproducibility?

Yes

Is the study design appropriate and is the work technically sound?

Yes

Are the conclusions drawn adequately supported by the results?

Partly

Are sufficient details of methods and analysis provided to allow replication by others?

Yes

Reviewer Expertise:

Database, ontology, information retrieval

I confirm that I have read this submission and believe that I have an appropriate level of expertise to confirm that it is of an acceptable scientific standard.

# Response

**Published:** 2021-11-17

**Authors:** Haw Su Cheng .

The paper intended to show the advantage of blockchain to improve data access control for the healthcare domain. The goal is to provide a blockchain framework to resolve decentralised access and authentication control. However, in my opinion, the paper needs some clarifications in regards to data analysis and text:

- You said Algorithm 3 and 4 run on the same evaluation PC, but I didn't find Algorithm 4 in the text.

***Response:*** *Corrected* *.* *We have re-organised the algorithms (Algorithms 3 and 4) to ensure a clearer description*.

*......................................................................................................*

- Algorithm 3 hasn't been explained adequately, especially in lines 4-5; and returned values in lines 10 and 12, what is the difference between the two?

***Response:*** *Corrected. We have elaborated further in the description.*

*........................................................................................................*

- In the sense of statistical analysis and interpretation, you said the latency was getting lower because the volume of pending transactions grew high? This needs a clear explanation.

***Response*** *: Corrected. This analysis has been restructured and explained in detail in the revised version.*

  .......................................................................................................

- Add the point of blockchain framework to resolve decentralised access and authentication control in the discussions.

***Response*** *: Corrected. This has been addressed in the revised version.*

# Peer review: Reviewer response for version 2

**DOI:** 10.5256/f1000research.78587.r98039

**Published:** 2021-11-02

**Authors:** Huo Chong Ling

**Affiliations:** 1 Department of Electrical and Computer Engineering, Curtin University Malaysia, Miri, Malaysia

The authors have addressed all the comments. I have no further comments.

Is the work clearly and accurately presented and does it cite the current literature?

Yes

If applicable, is the statistical analysis and its interpretation appropriate?

Partly

Are all the source data underlying the results available to ensure full reproducibility?

Yes

Is the study design appropriate and is the work technically sound?

Yes

Are the conclusions drawn adequately supported by the results?

Yes

Are sufficient details of methods and analysis provided to allow replication by others?

Yes

Reviewer Expertise:

Security

I confirm that I have read this submission and believe that I have an appropriate level of expertise to confirm that it is of an acceptable scientific standard.

# Peer review: Reviewer response for version 1

**DOI:** 10.5256/f1000research.76499.r94763

**Published:** 2021-10-08

**Authors:** Huo Chong Ling

**Affiliations:** 1 Department of Electrical and Computer Engineering, Curtin University Malaysia, Miri, Malaysia

The authors proposed a decentralized network using blockchain technology to store and share health data securely by adopting a PKI model in their blockchain framework. The PKI model consists of content verification and authentication and secure access. Experiments were conducted to evaluate the performance of the proposed blockchain framework in terms of throughput, latency, scalability, and security, and compared to existing approaches in the literature. The experiments showed promising results, which indicate the feasibility of the framework to be used in the healthcare domain.

 However, there are a few doubts regarding the statistical analysis of the experimental results as follows:

1. Do the results obtained consist of the average of several runs or just one run?
2. Are the algorithms used in [4] and [5] implemented as well on the same PC that is used for the proposed approach? This is to ensure fair performance evaluation.
3. The latency results showed the average for the block size of 200-400. How about the latency results for the block size of 400 and above?

Other comments on the article are as follows:

- The year of publication for Yang *et al.* is 2019, but in your figures, it is shown as 2018.
- It's better to write "Proposed approach" instead of "My approach" in the figures.
- Reference 20 is not cited in the text.

Is the work clearly and accurately presented and does it cite the current literature?

Yes

If applicable, is the statistical analysis and its interpretation appropriate?

Partly

Are all the source data underlying the results available to ensure full reproducibility?

Yes

Is the study design appropriate and is the work technically sound?

Yes

Are the conclusions drawn adequately supported by the results?

Yes

Are sufficient details of methods and analysis provided to allow replication by others?

Yes

Reviewer Expertise:

Security

I confirm that I have read this submission and believe that I have an appropriate level of expertise to confirm that it is of an acceptable scientific standard, however I have significant reservations, as outlined above.

# Response

**Published:** 2021-10-22

**Authors:** Haw Su Cheng .

- Do the results obtained consist of the average of several runs or just one run?

***Author response:*** *Yes, between 1000 to 10,000 transactions were asynchronously sent to the blockchain server.* *The number of requests is set from 1000 to 10,000 transactions, with increments of 1000 transactions for every run.*

- Are the algorithms used in [4] and [5] implemented as well on the same PC that is used for the proposed approach? This is to ensure fair performance evaluation.

***Author response:*** *Yes, this was included in the proposed model access chaincodes, which record all ‘user’ activities, such as the record of shared data or information access history on the blockchain. Algorithm 3 is executed when transaction access is requested, and similarly, Algorithm 4 is executed for read/write transactions.*

-  The latency results showed the average for the block size of 200-400. How about the latency results for the block size of 400 and above?

***Author response*** *We have studied latency and throughput up to 600 blocks in the thesis presented for the work. Also, as stated in observations for all block sizes as for all block sizes, except for when the transaction is at 1000, we saw that latency of all approaches are lower.*

 Other comments on the article are as follows:

- The year of publication for Yang *et al.* is 2019, but in your figures, it is shown as 2018.

***Author response*** ***:** This has been adjusted in figures*

- It's better to write "Proposed approach" instead of "My approach" in the figures.

***Author response*** ***: **This has been adjusted in figures as advised*

- Reference 20 is not cited in the text.

***Author response*** ***:** Citation added.*
